# Defective i^6^A37 Modification of Mitochondrial and Cytosolic tRNAs Results from Pathogenic Mutations in TRIT1 and Its Substrate tRNA

**DOI:** 10.1371/journal.pgen.1004424

**Published:** 2014-06-05

**Authors:** John W. Yarham, Tek N. Lamichhane, Angela Pyle, Sandy Mattijssen, Enrico Baruffini, Francesco Bruni, Claudia Donnini, Alex Vassilev, Langping He, Emma L. Blakely, Helen Griffin, Mauro Santibanez-Koref, Laurence A. Bindoff, Ileana Ferrero, Patrick F. Chinnery, Robert McFarland, Richard J. Maraia, Robert W. Taylor

**Affiliations:** 1 Wellcome Trust Centre for Mitochondrial Research, Institute for Ageing and Health, The Medical School, Newcastle University, Newcastle upon Tyne, United Kingdom; 2 Intramural Research Program, NICHD, NIH, Bethesda, Maryland, United States of America; 3 Wellcome Trust Centre for Mitochondrial Research, Institute for Genetic Medicine, The Medical School, Newcastle University, Newcastle upon Tyne, United Kingdom; 4 Department of Life Sciences, University of Parma, Parma, Italy; 5 Department of Neurology, Haukeland University Hospital, Bergen, Norway; 6 Department of Clinical Medicine, University of Bergen, Bergen, Norway; Max Planck Institute for Biology of Ageing, Germany

## Abstract

Identifying the genetic basis for mitochondrial diseases is technically challenging given the size of the mitochondrial proteome and the heterogeneity of disease presentations. Using next-generation exome sequencing, we identified in a patient with severe combined mitochondrial respiratory chain defects and corresponding perturbation in mitochondrial protein synthesis, a homozygous p.Arg323Gln mutation in *TRIT1*. This gene encodes human tRNA isopentenyltransferase, which is responsible for i^6^A37 modification of the anticodon loops of a small subset of cytosolic and mitochondrial tRNAs. Deficiency of i^6^A37 was previously shown in yeast to decrease translational efficiency and fidelity in a codon-specific manner. Modelling of the p.Arg323Gln mutation on the co-crystal structure of the homologous yeast isopentenyltransferase bound to a substrate tRNA, indicates that it is one of a series of adjacent basic side chains that interact with the tRNA backbone of the anticodon stem, somewhat removed from the catalytic center. We show that patient cells bearing the p.Arg323Gln *TRIT1* mutation are severely deficient in i^6^A37 in both cytosolic and mitochondrial tRNAs. Complete complementation of the i^6^A37 deficiency of both cytosolic and mitochondrial tRNAs was achieved by transduction of patient fibroblasts with wild-type TRIT1. Moreover, we show that a previously-reported pathogenic m.7480A>G mt-tRNA^Ser(UCN)^ mutation in the anticodon loop sequence A36A37A38 recognised by TRIT1 causes a loss of i^6^A37 modification. These data demonstrate that deficiencies of i^6^A37 tRNA modification should be considered a potential mechanism of human disease caused by both nuclear gene and mitochondrial DNA mutations while providing insight into the structure and function of TRIT1 in the modification of cytosolic and mitochondrial tRNAs.

## Author Summary

Mitochondrial disorders are clinically diverse, and identifying the underlying genetic mutations is technically challenging due to the large number of mitochondrial proteins. Using high-throughput sequencing technology, we identified a disease-causing mutation in the *TRIT1* gene. This gene encodes an enzyme, tRNA isopentenyltransferase, that adds an *N^6^* ^-^isopentenyl modification to adenosine-37 (i^6^A37) in a small number of tRNAs, enabling them to function correctly during the synthesis of essential mitochondrial proteins. We show that this mutation leads to severe deficiency of tRNA-i^6^A37 in the patient's cells that can be rescued by introduction of the wild-type TRIT1 protein. A deficiency in oxidative phosphorylation, the process by which energy (ATP) is generated in the mitochondria, leads to a mitochondrial disease presentation. Introducing the mutant protein into model yeast species and measuring the resulting impairment provided further evidence of the pathogenic effect of the mutation. Additional studies investigating a previously reported pathogenic mutation in a mitochondrial tRNA gene demonstrated that a mutation in a substrate of TRIT1 can also cause a loss of the modification, providing evidence of a new mechanism causing mitochondrial disease in humans.

## Introduction

Mitochondrial diseases are characterised by biochemical defects in oxidative phosphorylation (OXPHOS) enzyme activity and arise as a consequence of nuclear- or mitochondrial-encoded gene mutations [1]. Generalised disorders of mitochondrial protein synthesis resulting in OXPHOS defects are increasingly reported as causing a clinically heterogeneous group of neonatal and infantile mitochondrial disease presentations associated with isolated or multi-organ involvement [2]. The advent of whole exome capture and sequencing technologies has revolutionised the molecular diagnosis of this patient group [3]; the molecular disease mechanism can implicate nuclear gene products involved in mitochondrial DNA (mtDNA) replication, synthesis and repair [4], mitochondrial aminoacyl tRNA synthetases [5], mitochondrial translation elongation and release factors [6], structural ribosomal proteins and assembly factors, and enzymes involved in mt-RNA modification [7].

Post-transcriptional modification of tRNAs is crucial for folding, stability and function in deciphering the genetic code during translation. Modifications of cytosolic-tRNAs (cy-tRNA) and mt-tRNAs occur notably at nucleotide positions 34 and 37 in the anticodon loop (ACL), serving to promote translational fidelity and efficiency by optimising codon-anticodon fit within the ribosome [8]. The homologous tRNA isopentenyltransferases (IPTases) are conserved from bacteria to humans and introduce an evolutionarily ancient modification, an isopentenyl group onto *N^6^* of adenine at position 37 (i^6^A37). In bacteria, i^6^A37 is further modified by methylthiolation to ms^2^i^6^A37 which uses its methyl-sulphur group to stabilise the intrinsically weak A–U pairing between anticodon A36 and the first base of UNN codons [9]. However, the methylthiolation enzymes are not present in eukaryotes, leaving i^6^A37 without further modification.

To date, functional analysis in eukaryotes comes from studies in yeast which have shown that i^6^A37 promotes translational efficiency and fidelity in a codon-specific manner cognate with the i^6^A37-tRNAs [10]. The presence of i^6^A37 increases the specific activity of a tRNA for its codon about four-fold in *S. pombe* [10]. Prior work on orthologues including MiaA (*E. coli*), Tit1 (*S. pombe*), Mod5 (*S. cerevisiae*), and TRIT1 (human) has revealed specificity for subsets of cy- and mt-tRNAs that bear the single-stranded anticodon loop recognition sequence, ‘A36-A37-A38’, although this motif alone is not always sufficient ([11] and refs therein). However, it has also become clear that due to sequence variability in the tRNAs and the specificities of the transferases, different species contain different subsets of i^6^A37-modified tRNAs [10], [12]. Determining the subsets of specific mRNAs that are sensitive to i^6^A37 deficiency and how this contributes to phenotype is a contemporary challenge [10].

We used whole exome sequencing to identify a homozygous p.Arg323Gln mutation in the *TRIT1* gene that segregates within a consanguineous UK-Pakistani family in which affected children present with encephalopathy and myoclonic epilepsy due to multiple OXPHOS deficiencies in skeletal muscle. We confirm that this mutation is responsible for a severe deficiency in the i^6^A37 content of cy- and mt-tRNAs, as it can be reversed by rescue with wild type TRIT1 in the patient's fibroblasts. We show that TRIT1 is targeted to mitochondria and provide evidence in both humans and yeast that this gene is required for efficient mitochondrial function. Furthermore, we have demonstrated that a previously-reported pathogenic A38G mutation of mt-tRNA^Ser(UCN)^, causes i^6^A37 deficiency, strengthening the conclusion that TRIT1-related human disease can arise from mutation of either the enzyme or its tRNA substrate.

## Results

### Histochemical and biochemical analyses identified a generalised disorder of mitochondrial protein synthesis

**Figure 1:**
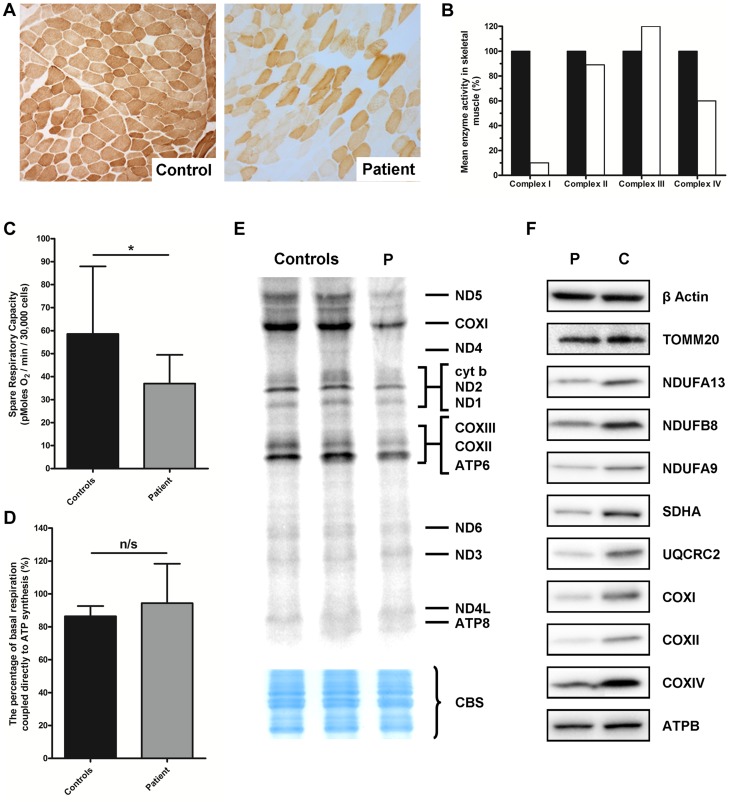
Identification of a mitochondrial respiratory chain deficiency and defective mtDNA translation. **A**) Cytochrome *c* oxidase (COX) histochemical reactivity revealed a mosaic of COX-deficiency in patient skeletal muscle compared to control. **B**) The assessment of individual respiratory chain enzyme activities identified a combined OXPHOS deficiency affecting complexes I and IV in skeletal muscle from the proband. The mean activity measured in 25 controls was set at 100%. **C**) Patient fibroblasts (grey) are less capable of responding to stress in comparison to control fibroblasts (black), as measured by the spare respiratory capacity. *: P<0.05. **D**) The coupling efficiency of ATP synthesis and respiration, and therefore the level of proton leak, is not decreased in patient fibroblasts (grey) compared to controls (black). The error bars displayed on each graph indicate standard deviation. **E**) *In vitro* metabolic labelling of mitochondrial translation in patient fibroblasts (P) showed a generalised decrease in translation activity, with the subunits of Complex I (notably ND5) and Complex IV (notably COXI) most substantially decreased. Even loading was confirmed by Coomassie blue staining (CBS). **F**) Immunoblotting demonstrated a generalised decrease in the level of individual subunits from respiratory chain complexes I–V (normalised to β-actin) in patient fibroblasts, whilst the mitochondrial marker, TOMM20 was unchanged.

We investigated a family with clinical indications of mitochondrial disease in two affected children. A skeletal muscle biopsy (subject II-3, detailed clinical report in Text S1) showed normal morphology and a mosaic pattern of cytochrome *c* oxidase (COX) deficiency (Figure 1A), which can be associated with mutations in nuclear genes involved with mtDNA translation and maintenance or mtDNA mutations. We also observed biochemical evidence of a mitochondrial respiratory chain deficiency involving complexes I (10% of controls) and IV (60% of controls), with apparent sparing of complex II and III activity (Figure 1B). Together these data confirmed the presence of a combined OXPHOS deficiency.

Micro-scale oxygraphy analysis provided evidence of mitochondrial respiratory dysfunction in patient fibroblasts (Figure 1C and D). Basal oxygen consumption rate (OCR) was significantly decreased (P = 0.0451) in the patient compared to controls, as was maximal OCR (P = 0.0078). The spare respiratory capacity (SRC) (Figure 1C) was significantly reduced (P = 0.0102) in patient cells whilst the coupling efficiency of ATP synthesis to respiration, a measure of proton leak, was unchanged (Figure 1D).

*In vitro* metabolic labelling of mitochondrial translation identified a generalised decrease in the synthesis of mtDNA-encoded proteins with particularly notable loss of ND1 and ND5 of Complex I, CYTB of Complex III and COXI, COXII and COXIII of Complex IV (Figure 1E). This was supported by immunoblotting, which revealed decreased steady-state levels of mtDNA-encoded OXPHOS components in the patient fibroblasts (Figure 1F) and a moderate decrease in SDHA protein levels which was surprising given that complex II activity in skeletal muscle was normal (Figure 1F). Levels of TOMM20 were unchanged in patient cells confirming a specific defect of OXPHOS protein synthesis rather than general loss of mitochondrial proteins.

### Exome sequencing identified a mutation in *TRIT1*

**Figure 2:**
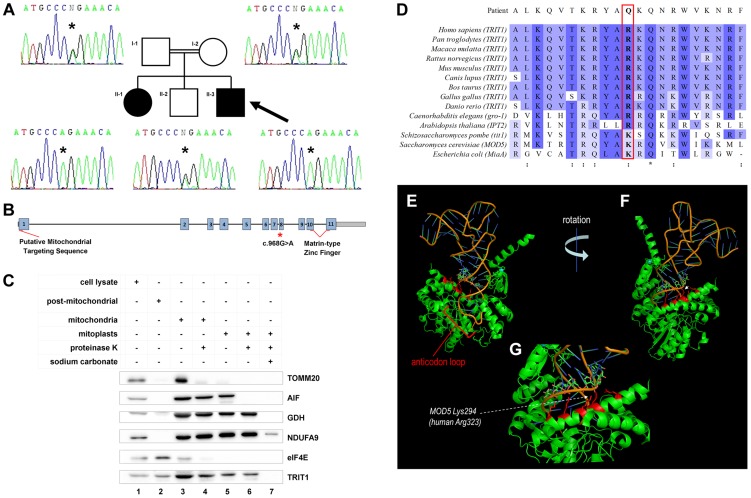
A *TRIT1* mutation segregates with disease and disrupts a conserved tRNA-binding basic side-chain. **A**) Targeted resequencing of *TRIT1* confirmed that the proband (II-3; arrow), and his clinically affected sister (II–1) are homozygous for the c.968G>A *TRIT1* mutation, while his unaffected older brother (II–2) and both of his parents (I–1 and I–2) are heterozygous carriers. **B**) The *TRIT1* mutation is located in exon 8, whilst there is a putative mitochondrial targeting sequence in exon 1 and a matrin-type zinc finger domain spanning exons 10 and 11. **C**) The mitochondrial sub-localisation of TRIT1 is demonstrated by sub-fractionation and immunoblotting, using markers for each sub-fraction to confirm there was no contamination: TOMM20 (mitochondrial outer membrane), AIF (mitochondrial intermembrane space), GDH (mitochondrial matrix), NDUFA9 (mitochondrial inner membrane) and eIF4E (cytosol). TRIT1 localized with eIF4E in the cytosol (lane 2) and showed the same profile as GDH (lanes 3–6), but was undetectable in the inner mitochondrial membrane fraction (lane 7). A total of 40 µg protein was loaded for each sample, and all mitochondrial subfractions were prepared from the same mitochondrial lysate. **D**) Clustal Omega alignment of the TRIT1 protein and known orthologs revealed that the affected amino acid (p.Arg323) is conserved in each species excluding *S. pombe, S. cerevisiae* and *E. coli*, where the equivalent amino acid is lysine, which has similar electrochemical properties (:). Asterisks (*) indicate completely conserved residues. **E–G**) The co-crystal structure of Mod5 bound to a substrate tRNA (based on [14]) shows the interaction of the tRNA backbone (nucleotides 27–29) with an extended α-helix in which are located multiple basic side chains (indicated in red) of the enzyme including that corresponding to the mutated position (Lys294).

Having excluded mtDNA rearrangements, copy number abnormalities and point mutations (Table S1), we employed whole exome sequencing of both affected siblings to elucidate a potential genetic basis of the defect. This analysis identified 3970 novel homozygous protein altering variants shared between siblings (Table S2), of which 40 were rare (Minor Allele Frequency <0.01). Based on predicted mitochondrial localisation and an autosomal recessive inheritance pattern, variant filtering identified a single candidate homozygous missense mutation shared by both affected siblings in *TRIT1* (c.968G>A predicting p.Arg323Gln). This mutation was predicted to be pathogenic by PolyPhen-2 (http://genetics.bwh.harvard.edu/pph2/) with a score of 0.999. Targeted resequencing of the proband and familial relatives confirmed the homozygous mutation in the affected siblings and demonstrated disease segregation as both parents and an unaffected sibling were heterozygous carriers (Figure 2A). Importantly, the *TRIT1* c.968G>A variant was not observed by the 1000 Genomes Project, the NHLBI Exome Sequencing Project nor a panel of 120 ethnically-matched control chromosomes (data not shown).

The p.Arg323Gln mutation occurs in exon 8 of the *TRIT1* gene, which also has a putative mitochondrial targeting sequence in exon 1 and a matrin-type zinc finger domain contributed by exons 10 and 11 (Figure 2B). Mitochondrial targeting of TRIT1 is supported by prediction using the freely available online tool, MitoProt II (http://ihg.gsf.de/ihg/mitoprot.html) [13], with a confidence of 94%. Both cytosolic and mitochondrial localization is predicted by other available databases, as is also the case for its homologs Mod5, Tit1, and GRO-1. Indeed, immunoblotting of whole cell extracts and isolated mitochondrial subfractions confirmed that TRIT1 was present in the cytosolic fraction (Figure 2C, lane 2) and also detectable in proteinase K-treated mitoplasts (lane 6), consistent with the enzyme being present in the mitochondrial matrix, where tRNA molecules and the translation apparatus are active during protein synthesis. However, at this stage we cannot exclude the possibility that a significant fraction of the cytosolic portion of TRIT1 may be associated with the outer mitochondrial membrane.

The corresponding position of the affected amino acid, p.Arg323, is occupied by a basic side chain in all homologues from a range of species (Figure 2D), whereas glutamine at this position in the proband is polar but uncharged. Based on the available Mod5-tRNA co-crystal structure [14] (Figure 2E–G), the position and chemical nature of the mutation was not expected to affect mitochondrial localization, general solubility or gross structural alterations of the enzyme. In Mod5 this position is occupied by p.Lys294 whose basic side chain extends from an α-helix that lies adjacent to, but pointing away from, the catalytic site containing A37 [14]. Both this and adjacent conserved basic side chains comprise part of a series of residues on the same side of the α-helix, including Arg298 (also arginine in TRIT1) which are involved in binding the phosphate groups of nucleotides 27–29 of the AC stem (Figure 2E–G) [14]. Based on this we suspected that the mutation would not impair catalytic activity *per se* but might impair proper binding of the enzyme to its tRNA substrates. However, since this mutation would appear to affect only one of a series of contacts with the surface of the tRNA backbone, it was not necessarily expected to cause a severe deficiency of isopentenyltransferase activity.

### The *TRIT1* p.Arg323Gln mutation severely impairs i^6^A modification activity

**Figure 3:**
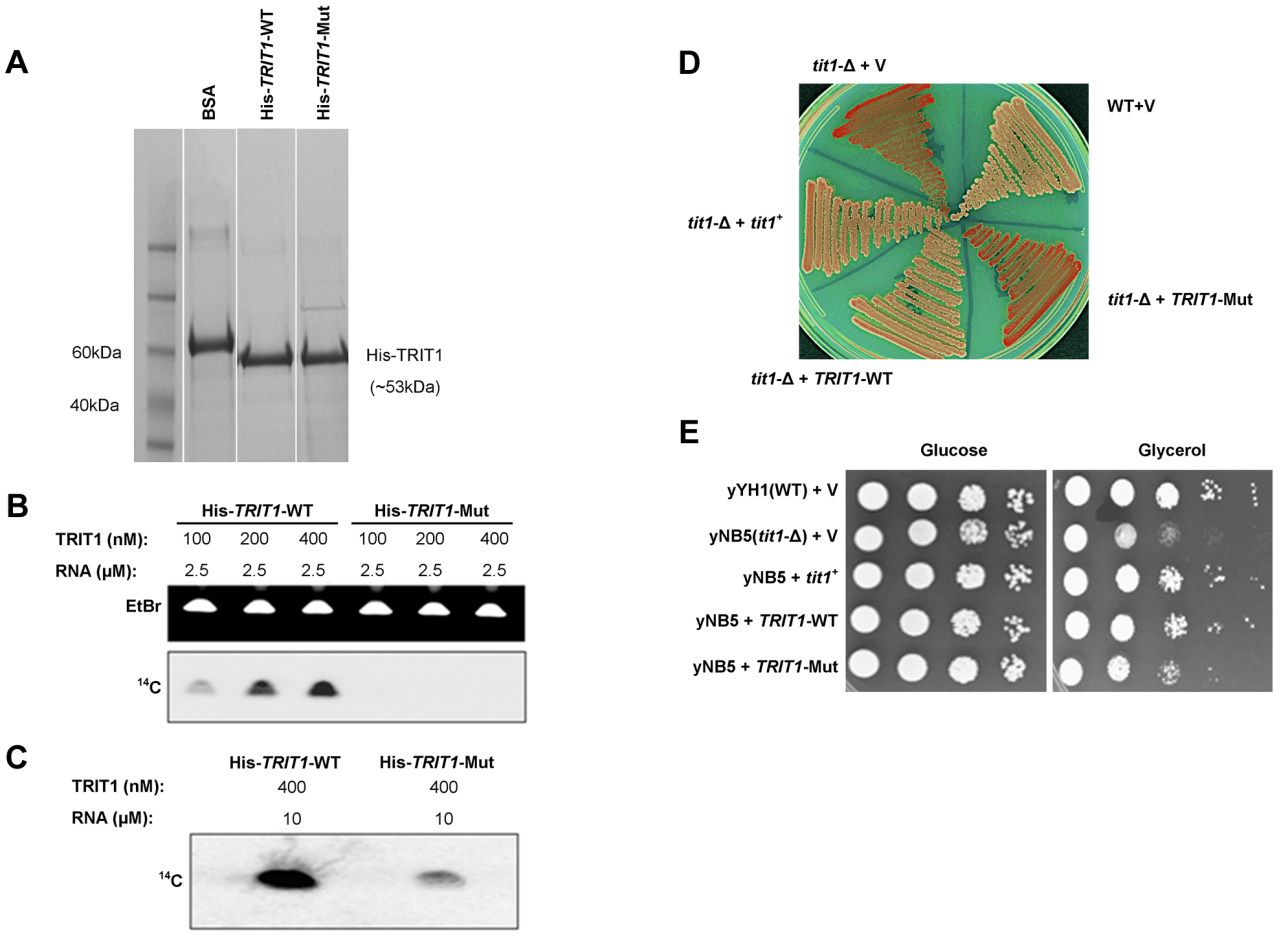
Mutant TRIT1 has decreased *in vitro* activity and cannot complement *tit1*-Δ defects in *S. pombe*. **A**) A Coomassie-blue stained SDS polyacrylamide gel confirmed the parallel purification of His-tagged TRIT1-WT and His-tagged TRIT1-Mut from *E. coli*. **B**) Loss of the isopentenyltransferase activity of TRIT1 carrying the p.Arg323Gln mutation was demonstrated by an *in vitro* assay using varying concentrations (nM) of His-tagged TRIT1-WT and TRIT1-Mut as well as a standard amount of RNA substrate (2.5 µM) [11]. **C**) Mutant TRIT1 activity was shown to be very low rather than absent by repeating the assay using 10 µM RNA and 400 nM of protein. **D**) Wild-type (yYH1) and *tit1-Δ* (yNB5) strains of the fission yeast *Schizosaccharomyces pombe* were transformed with an empty vector (+V), the *S. pombe* tRNA isopentenyltransferase (+*tit1^+^*), wild-type human *TRIT1* (+*TRIT1*-WT) or mutant human *TRIT1* (+*TRIT1*-Mut) and plated onto media containing limited adenine to assay for the loss of function of tRNA^Ser(UCA)^ due to lack of isopentenyl modification. *tit1-*deleted yeast carrying the empty vector or mutant *TRIT1* showed no recovery of tRNA^Ser(UCA)^ function (red colonies), but knock-down yeast carrying wild-type *TRIT1* or *tit1^+^* showed recovery of tRNA^Ser(UCA)^ activity (white colonies) similar to wild-type yeast. **E**) Transformation of *tit1-Δ* yeast with wild-type *TRIT1* or *tit1^+^,* but not the empty vector or mutant *TRIT1*, could also complement the respiratory deficiency illustrated by slow growth on glycerol compared to growth on glucose; spots reflect 10-fold serial dilutions of the same amounts of cells as determined by OD_600_.

Purified recombinant TRIT1 was previously used to examine i^6^A modification activity *in vitro* using an established assay employing synthetic RNA that matches the anticodon stem loop (ASL) of a substrate tRNA [11]. By this assay, the isopentenyl group of DMAPP (dimethylallyl pyrophosphate) is transferred to *N^6^* of A37 in substrate tRNAs by the IPTase TRIT1 ([11] and references therein). His-tagged TRIT1-WT and His-tagged TRIT1-Arg323Gln were purified from *E. coli* in parallel and compared by gel electrophoresis (Figure 3A). The modification activity of mutant TRIT1 was negligible relative to that of wild-type TRIT1 using the standard assay (Figure 3B). We reasoned that if the mutation led to decreased affinity for its substrate, as suggested by the co-crystal structure of Mod5-tRNA, we might be able to obtain activity by increasing substrate concentration. Some activity of mutant TRIT1 could indeed be observed by increasing the concentration of the RNA substrate 4-fold, but even under these conditions it was much less active than the wild-type TRIT1 (Figure 3C). Although increasing the concentrations of enzyme and substrate further was technically-limited in these reactions, the data suggest that higher activity might be achieved with higher concentrations.

### Mutant TRIT1 fails to complement activity of a codon-specific cytoplasmic translational reporter or a mitochondrial respiratory growth defect in a *tit1*-knockout strain of *S. pombe*

A previously characterized *S. pombe* strain with a deletion of the *tit1* ^+^ gene (a homologue of *TRIT1*), yNB5, exhibits two distinct phenotypes [10], [11] that were examined for their sensitivity to wild-type TRIT1 and the p.Arg323Gln TRIT1 mutant. The first phenotype is manifested by a red-white colony colour assay, that monitors tRNA^Ser(UCA)^-mediated suppression of a UGA nonsense mutation in *ade6-704*. This *in vivo* assay reports on the codon-specific translational activity of the suppressor-tRNA^Ser(UCA)^ to decode the *ade6-704* UGA codon, which was previously shown to be highly dependent on i^6^A37 [10], [11]. In this assay, absence of i^6^A37 decreases the translational activity of the suppressor-tRNA and the cells accumulate red pigment [11]. The yNB5 strain (*tit1*-Δ) transformed with the empty vector is red as expected, whilst the yYH1 strain (*tit1* **^+^**) is white. yNB5 transformed with either wild-type *TRIT1* or wild-type *tit1* ^+^ are white, indicating successful complementation, whilst yNB5 transformed with mutant *TRIT1* is red (Figure 3D).

The second phenotype of the yNB5 strain is slow growth in glycerol, which is a manifestation of mitochondrial respiratory dysfunction. This growth defect could be rescued by *tit1* ^+^ but not by a catalytically debilitated mutant-*tit1* carrying a point mutation [10]. The positive control, yYH1 (*tit1* **^+^**), grows well when transformed with an empty vector, but yNB5 transformed with an empty vector grows relatively poorly on glycerol. Transformation with wild-type *tit1* ^+^ or wild-type *TRIT1* rescued the growth defect of yNB5. However, whilst growth on glycerol after transformation with the mutant *TRIT1* was slightly better than with the empty vector, rescue was less complete compared to wild-type TRIT1 (Figure 3E). This partial rescue may reflect a low level of i^6^A37 modification activity by the mutant *TRIT1* enzyme.

### Modelling of the mutation in *S. cerevisiae* demonstrates a respiratory deficiency

We also generated a strain of *S. cerevisiae* with knock-out *MOD5* (a homologue of *TRIT1*) which was transformed with either wild-type *MOD5*, an empty vector, *mod5* ^K294R^ (humanised *MOD5*, which carries the Lys294Arg mutation) or *mod5* ^K294Q^ (mutant *MOD5*, which carries the Lys294Gln mutation). As observed in *S. pombe*, *mod5-Δ* yeast transformed with *mutant MOD5* showed a reduced growth rate in a oxidative carbon source such as ethanol compared to *mod5-Δ* yeast transformed with either wild-type *MOD5* or humanised *MOD5* (Figure S1A). Oxidative growth defects were due to reduced respiratory activity since *mod5-Δ* strain transformed with an empty vector showed a significantly decreased respiration rate (P  =  0.0002) in comparison to yeast transformed with wild-type *MOD5,* whilst mutant *MOD5* failed to rescue the respiration rate of the transformed yeast as efficiently as wild-type or humanised *MOD5* (Figure S1B).

### The i^6^A37 modification is severely decreased in tRNAs from patient-derived fibroblasts carrying the p.Arg323Gln mutation

**Figure 4:**
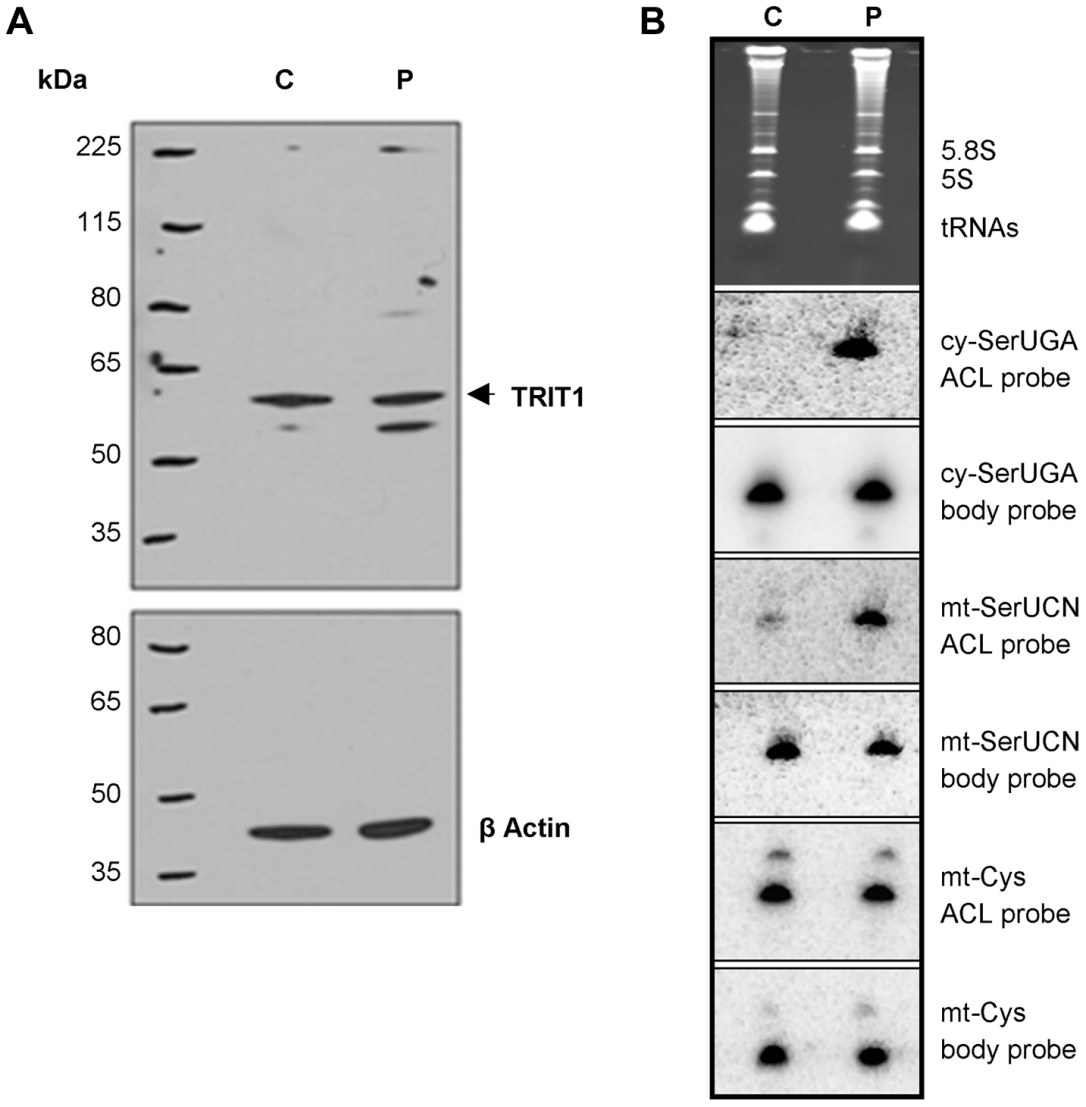
The *TRIT1* mutation disrupts modification activity on cytosolic and mitochondrial tRNAs but not enzyme abundance. **A**) No decrease in the levels of the native TRIT1 protein in patient fibroblasts was observed by immunoblotting (using β-actin as a loading control) **B**) The isopentenyl modification status of both mitochondrial (mt-) and cytosolic (cy-) tRNAs in patient fibroblasts (lane P) compared to controls (lane C); by this approach a positive signal is due to lack of isopentenyl modification as detected by an anticodon loop (ACL) probe (the bulky modification on the N of adenine blocks base pairing with the probe, such that no signal for cy-tRNA^Ser(UGA)^ with the ACL probe indicates efficient modification in the control cells [11]); a body probe to a different region of the same tRNA is used as a control for calibration and calculation of steady-state levels. Each panel shows hybridisation of the same blot with a different probe as indicated to the right. The cytosolic tRNA^Ser(UGA)^ is poorly modified in patient fibroblasts (strong ACL probe signal), but tRNA^Ser(UGA)^ steady-state levels are unchanged. Mt-tRNA^Ser(UCN)^ is also poorly modified in patient fibroblasts, although a small pool of mt-tRNA^Ser(UCN)^ in control fibroblasts lacks the modification. The modification appears to be influential on mt-tRNA^Ser(UCN)^ stability, as steady-state levels are decreased by 40% in the patient. The non-substrate mt-tRNA^Cys^ was probed as a control.

Immunoblotting of TRIT1 in fibroblasts from the proband demonstrated no significant loss of protein levels in comparison to control fibroblasts, using β-actin as a loading control (Figure 4A). A second, smaller band that was barely detectable in the control cell extract but more abundant in the patient cell extract was not always reproducible, likely reflecting nonspecific protein degradation. Notably, our evidence indicates that mitochondrial TRIT1 is the same molecular weight as the major band observed in the extracts (see Figure 2C). We next examined the *in vivo* levels of mitochondrial and cytosolic tRNA-i^6^A37 in patient and control fibroblasts using the **P**ositive **H**ybridisation in the **A**bsence of i**^6^**A (PHA6) assay [11]. As described previously, in the PHA6 assay, strong binding of the ACL probe occurs only in the absence of i^6^A37, whilst body probes (BP) efficiently bind the tRNA whether or not i^6^A37 is present and were used to indicate relative levels of the tRNAs [11]. Equal loading of the RNA was confirmed by ethidium bromide imaging of the gel (Figure 4B, upper panel) and by hybridization with appropriate BPs.

**Figure 5:**
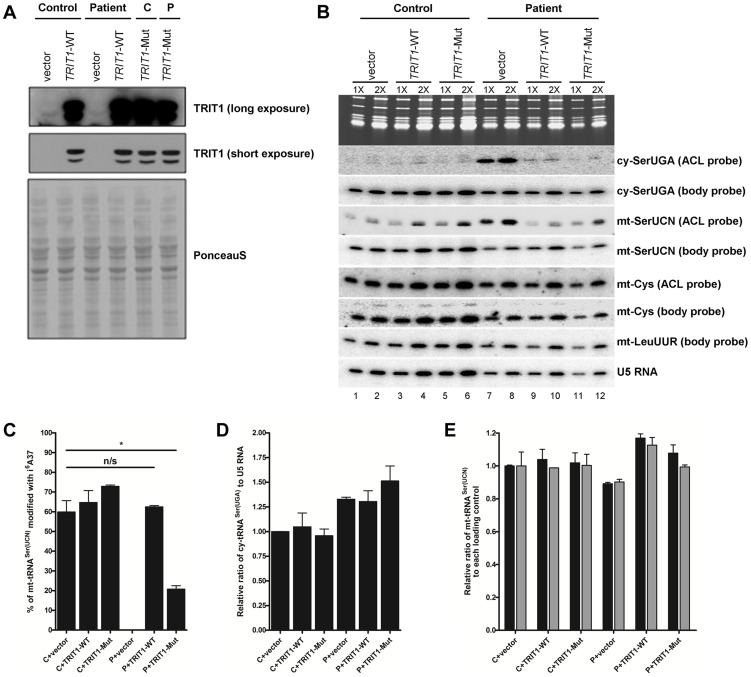
TRIT1 activity in patient fibroblasts is rescued by transduction with wild-type *TRIT1*. **A**) Immunoblotting of the TRIT1 protein in patient and control fibroblasts after transduction with an empty vector, wild-type *TRIT1* (*TRIT1*-WT) or mutant *TRIT1* (*TRIT1-*Mut) and selection with puromycin demonstrated overexpression, whilst Ponceau S staining confirmed even loading. **B**) The PHA6 assay was performed on patient and control fibroblasts transduced with wild-type *TRIT1*, mutant *TRIT1* or an empty vector, for both mitochondrial (mt-) and cytosolic (ct-) tRNAs serine. Loading controls are provided by U5 RNA as well as two mitochondrial non-TRIT1 substrates, mt-tRNA^Cys^ and mt-tRNA^Leu(UUR)^. Each sample was run in duplicate with 3 µg (1X) and 6 µg (2X) of RNA as indicated above the lanes. Transduction of patient fibroblasts with both wild-type and mutant TRIT1 rescued the i^6^A modification of cytosolic tRNA^Ser(UGA)^. **C**) However, only transfection with wild-type TRIT1 was able to efficiently rescue the i^6^A modification of mt-tRNA^Ser(UCN)^. *: p<0.05. **D**) The steady-state level of cytosolic tRNA^Ser(UGA)^, calculated using the body probe and the probe to U5 RNA, was found to be increased in all three transduced patient fibroblasts, although this up-regulation was found to be non-specific for cy-tRNA^Ser(UGA)^ (data not shown). **E**) The steady-state level of mt-tRNA^Ser(UCN)^ was shown to be recovered by transduction with wild-type TRIT1 and to a lesser extent, mutant TRIT1 (using the body probe and the probes for U5 RNA (black columns) and mt-tRNA^Cys^ (grey columns)). The error bars indicate the difference between the duplicate determinations.

Both cy-tRNA^Ser(UGA)^ and mt-tRNA^Ser(UCN)^ had considerably decreased i^6^A37 modification in patient fibroblasts compared to control. In contrast, there was no difference in the ACL probing of mt-tRNA^Cys^, which does have a A36A37A38 target site for TRIT1 but is not modified, consistent with previous results [12]. The cy-tRNA^Ser(UGA)^ appears to be fully modified in control fibroblasts (undetectable with ACL probe) consistent with prior results using HeLa cells [12] but largely unmodified in patient fibroblasts (Figure 4B). The mt-tRNA^Ser(UCN)^ shows a significant, albeit decreased difference in the ACL signal between control and patient compare to cy-tRNA^Ser(UGA)^. This is due in part to a significant fraction of unmodified mt-tRNA^Ser(UCN)^ in the control cells, again consistent with prior results using HeLa cells [12]. This suggests that wild-type TRIT1 is only partially active on mt-tRNA^Ser(UCN)^ in control fibroblasts. The more similar mt-tRNA^Ser(UCN)^ ACL signals in control and patient fibroblasts is also due in part to a significantly lower amount of the overall level of mt-tRNA^Ser(UCN)^ in the patient, as revealed by the mt-tRNA^Ser(UCN)^ BP. We note that whilst cy-tRNA^Ser(UGA)^ showed similar steady-state levels in control and patient cells, the mt-tRNA^Ser(UCN)^ showed a 40% decrease in steady-state level (Figure 4B, quantification not shown but see Figure 5E), calculated using the BPs of mt-tRNA^Ser(UCN)^ and mt-tRNA^Cys^ as internal calibration standards [10].

### Wild-type TRIT1 rescues the i^6^A modification in patient fibroblasts

Attempts to rescue the i^6^A37 hypomodification observed in patient fibroblasts were met with significant technical challenges that produced variable levels of transient transfection efficiency and ectopic TRIT1 and therefore only partial rescue of the i6A37 hypomodification (data not shown). We therefore used a retrovirus vector-based transduction approach to optimize the percentage of cells expressing ectopic TRIT1. Transduction of control and patient fibroblasts resulted in very high levels of overexpression as compared to the empty vector (Figure 5A), allowing us to examine the i^6^A37 content of tRNAs from patient cells (Figure 5B). Importantly, wild-type *TRIT1* completely reversed the cy-tRNA^Ser(UGA)^ hypomodification defect in the patient cells, whereas the empty vector did not, providing strong evidence that the native endogenous mutant TRIT1 protein is responsible for the hypomodification (Figure 5B, compare lanes 7 & 8 with 9 & 10). Given the very high overexpression of TRIT1 in these cells (Figure 5A), it was not surprising based on our mutation structure analysis, *in vitro* modification results and partial rescue of slow growth in glycerol by the mutant TRIT1 protein, that mutant TRIT1 was no less efficacious than wild-type TRIT1 in rescuing the i^6^A37 hypomodification of cy-tRNA^Ser(UGA)^ in patient cells (Figure 5B).

In notable contrast to the rescue of cy-tRNA^Ser(UGA)^ hypomodification, the hypomodification of mt-tRNA^Ser(UCN)^ was rescued more efficiently by wild-type TRIT1 than mutant TRIT1 (Figure 5B). Moreover, restoration of i^6^A37 to mt-tRNA^Ser(UCN)^ was specifically associated with an overall increase in the steady state levels of this tRNA as reflected by the mt-tRNA^Ser(UCN)^ BP (Figure 5B; compare lanes 7 and 8 with 9 and 10). Quantification of independent triplicate sample sets revealed that whilst wild-type TRIT1 could effectively recover the i^6^A37 modification level of mt-tRNA^Ser(UCN)^ to that observed in the control fibroblasts, ∼60% (P = 0.6286), mutant TRIT1 was significantly less efficient, at ∼20% (P = 0.0146) (Figure 5C).

### Wild-type TRIT1 promotes accumulation of mt-tRNA^Ser(UCN)^

Using U5 snRNA as a loading control and mt-tRNA^Cys^ and mt-tRNA^Leu(UUR)^ as non-substrate mitochondrial controls, the steady-state levels of both cy-tRNA^Ser(UGA)^ (Figure 5D) and mt-tRNA^Ser(UCN)^ (Figure 5E) (calculated using BPs) in transduced fibroblasts were determined. Curiously, cy-tRNA^Ser(UGA)^ levels relative to U5 RNA were reproducibly found to be significantly higher in patient fibroblasts as compared to the control cells, regardless of whether the transducing vector encoded *TRIT1* or not (Figure 5D). However, mt-tRNA^Ser(UCN)^ levels, which were relatively lower in patient fibroblasts, were more efficiently rescued by wild-type TRIT1 (elevated relative to U5: P = 0.0162) than by mutant TRIT1 (unchanged relative to U5: P = 0.2038) or the empty vector (decreased relative to U5: P = 0.0055) when compared to control fibroblasts transduced with empty vector (Figure 5E, black bars). This quantitative trend was more significant when calibrating the mt-tRNA^Ser(UCN)^ levels relative to the non-substrate control, mt-tRNA^Cys^ in the patient cells (Figure 5E, grey bars).

We tried various approaches to rescue the biochemical, respiratory and molecular phenotypes in the patient fibroblasts. However due to limitations associated with the manipulation and transfection of patient and control fibroblasts, we were unable to do so with either wild-type or mutant TRIT1 despite multiple attempts (not shown). It appears that the cells had become less dependent on and/or less expressive of respiratory function with passage and handling.

### The m.7480A>G mutation at position 38 in mt-tRNA^Ser(UCN)^ impairs i^6^A37 modification

**Figure 6:**
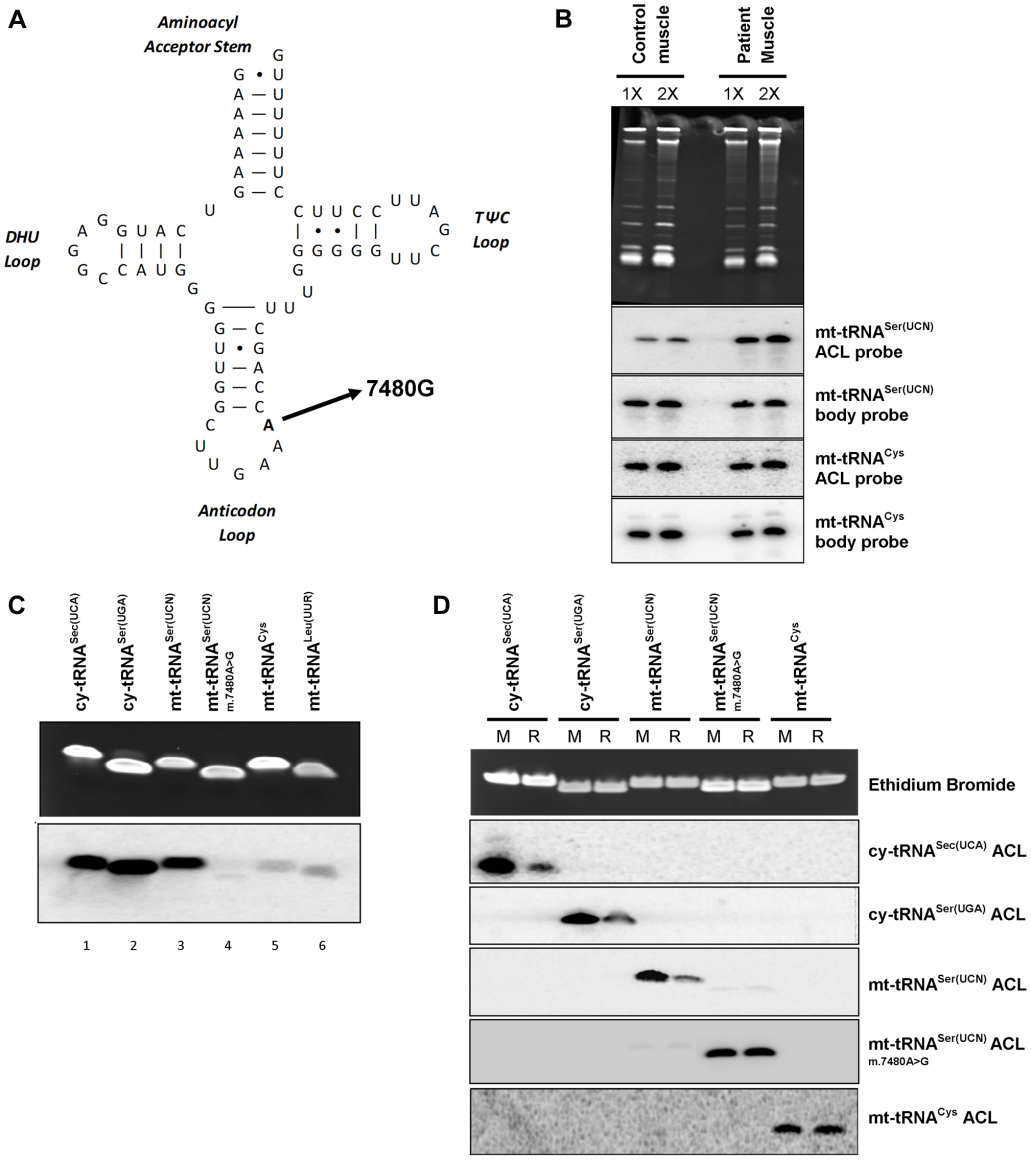
A mt-tRNA^Ser(UCN)^ point mutation, m.7480A>G, impairs i^6^A37 modification. **A**) The previously reported m.7480A>G mutation [15] is located at position 38 in the anticodon loop (ACL) of mt-tRNA^Ser(UCN)^, in the A36A37A38 recognition sequence of TRIT1. **B**) The i^6^A modification of position 37 is decreased (stronger binding of the mt-tRNA^Ser(UCN)^ double ACL probe) by ∼86% in patient skeletal muscle relative to control muscle. Binding of the ACL probe to the non-TRIT1 substrate, mt-tRNA^Cys^, confirmed even loading. The steady-state level of mt-tRNA^Ser(UCN)^ in patient skeletal muscle is 30% lower than in control muscle (calculated using the two body probes). Both control and patient samples were run in duplicate using 2 µg (1X) and 4 µg (2X) of RNA as indicated above the lanes. **C**) *In vitro* isopentylation assay using purified TRIT1, ^14^C-DMAPP, and synthetic minihelixes representing the anticodon stem loops (ASLs) of the tRNAs indicated above the lanes. The upper blot is an ethidium bromide-stained gel of the ASLs after *in vitro* reaction indicating even loading, whilst the lower blot is the autoradiograph obtained after 3 days of exposure. **D**) The PHA6 assay was validated and the loss of i^6^A37 modification in m.7480A>G mutant mt-tRNA^Ser(UCN)^ confirmed by *in vitro* modification of synthetic templates. For reaction samples (R), synthetic RNA minihelixes were *in vitro* modified using unlabeled-DMAPP and recombinant His-TRIT1. For mock-treated samples (M), all the components except the His-TRIT1 enzyme were added. After purification, the RNA samples were transferred to a membrane which was repeatedly hybridized, stripped and rehybridized with 5 different ^32^P-labeled ASL oligo probes as indicated to the right. Ethidium bromide staining of the gel confirmed equal loading of each pair of reactions (mock and reaction samples) for each synthetic ACL interrogated.

As noted in the Introduction, the tRNA substrates of all characterized isopentenyltransferases have an enzyme recognition sequence of A36A37A38 in their anticodon loops [11]. Thus we decided to further investigate a previously reported patient with mitochondrial myopathy [15] due to a pathogenic (m.7480A>G) mutation at position 38 in mt-tRNA^Ser(UCN)^, a substrate of TRIT1 (Figure 6A). The PHA6 assay using a double ACL probe that matches both the wild type and mutant mt-tRNA^Ser(UCN)^ (see Methods) performed on total RNA extracted from homogenised patient skeletal muscle showed reduced i^6^A37 modification of mt-tRNA^Ser(UCN)^, to ∼14% of control levels (Figure 6B, quantification not shown). Furthermore, the steady-state level of mt-tRNA^Ser(UCN)^ was also decreased by ∼30% in patient skeletal muscle compared to control (quantification not shown). Interestingly, when comparing control skeletal muscle to the previously described control fibroblasts (Figure 4B), it appears that skeletal muscle harbours relatively more mt-tRNA^Ser(UCN)^ lacking the i^6^A modification. The *in vitro* modification assay was also performed on several synthetic tRNA ASLs, substantiating our finding of a deficiency in modification activity (Figure 6C). Isopentenyl modification of wild-type mt-tRNA^Ser(UCN)^ (lane 3) *in vitro* is comparable to that observed for the positive controls, cy-tRNA^Sec(UCA)^ (Lane 1) and cy-tRNA^Ser(UGA)^ (Lane 2). However, the m.7480A>G mutation significantly abolishes *in vitro* activity of TRIT1 on mt-tRNA^Ser(UCN)^ (lane 4) to the level observed in the non-substrates, mt-tRNA^Cys^ (Lane 5) and mt-tRNA^Leu(UUR)^ (Lane 6). Hybridization of ASL probes to synthetic substrates modified *in vitro* by DMAPP was observed by the PHA6 assay to be substantially decreased compared to hybridization of the same probes to unmodified synthetic substrates (Figure 6D). Hybridization of ASL probes to the non-DMAPP substrate mt-tRNA^Cys^ was unaffected by treatment with TRIT1. Notably, hybridisation of ASL probes to mutant mt-tRNA^Ser(UCN)^ (m.7480A>G) was unchanged by the DMAPP modification reaction, confirming the loss of i^6^A37 modification in the mutant tRNA. These data validate the PHA6 assay for detection of changes in the i^6^A modification status of substrate mitochondrial and cytosolic tRNAs.

## Discussion

Here we describe the investigation of a consanguineous kindred in which affected children presented with encephalopathy and myoclonic epilepsy associated with a disorder of mitochondrial translation. Analysis of whole exome sequencing data indicated that this was due to a recessively-inherited p.Arg323Gln mutation in *TRIT1*, the gene encoding the tRNA isopentenyltransferase (IPTase) responsible for i^6^A modification at position 37 in the anticodon loop of a subset of tRNAs [16], including mt-tRNA^Ser(UCN)^, consistent with a previous report on i^6^A in bovine mt-tRNA^Ser(UCN)^ [17].

In addition to the TRIT1 mutation-associated disorder of mitochondrial dysfunction reported here, we also demonstrated that a m.7480A>G point mutation of mt-tRNA^Ser(UCN)^, previously reported as a cause of progressive mitochondrial myopathy [15], results in i^6^A37 hypomodification. In this case, the point mutation was in the TRIT1 sequence-specific recognition site, A36A37A38, of mt-tRNA^Ser(UCN)^. The convergence of two different mechanisms, one due to a mutation in the TRIT1 enzyme and the other to a mutation in its substrate mt-tRNA^Ser(UCN)^, that both cause i^6^A37 hypomodification of mt-tRNA^Ser(UCN)^ and mitochondrial myopathy, provide strong genetic evidence of the critical importance of i^6^A37 in mitochondrial translation. TRIT1 should therefore be added to the increasing list of genes encoding mitochondrial tRNA-modifying enzymes, including *MTU1* [18], *PUS1* [19], *MTO1* [20], *MTFMT* [21] and the various mitochondrial aminoacyl-tRNA synthetases [5], that have been associated with human disease. It is also worth noting that TRIT1 has been reported as a tumor suppressor and certain rare variant alleles are associated with poor survival from lung cancer in some ethnic groups [22], [23]; other mitochondrial-disease associated genes such as GRIM19 have also been implicated as a tumor suppressor [24]. However, it is not clear whether this is due to a relationship between cellular respiration and the mitochondrial function of TRIT1 in the lungs and/or the enzyme's cytosolic role. In addition, recent work has demonstrated an effect of Mod5 in tRNA-gene mediated gene silencing of RNA polymerase II promoters, suggesting a role for eukaryotic IPTases beyond their tRNA modification activity [25].

IPTases are conserved in sequence, structure and catalytic mechanism from bacteria to humans, particularly in the sequence surrounding the TRIT1 p.Arg323Gln mutation site. Indeed, in all of the IPTase sequences examined including *E. coli* MiaA, the targeted residue is either Arg or Lys. The mod5-i^6^A37-tRNA crystal structure shows that this residue comprises one of several basic side chains that contact the acidic backbone of the tRNA anticodon stem suggesting that the semi-conservative Arg to Gln mutation might compromise but not ablate enzyme activity. We therefore expected that any effect of the p.Arg323Gln mutation on TRIT1 activity would not be extreme and that the phenotype would be associated with a moderate decrease in tRNA i^6^A37 modification. However, to our surprise the mutation severely impaired the activity of the enzyme *in vitro* and caused a dramatic loss of i^6^A37 from both cy-tRNA^Ser(UGA)^ and mt-tRNA^Ser(UCN)^ in patient fibroblasts. It is important to note that our observation of i^6^A37 modification of cy-tRNA^Ser(UGA)^ contrasts with a previous study that could not detect this modification in human cy-tRNA^Ser(UGA)^ expressed in monkey-derived CV-1 cells [26].

In all eukaryotes examined, the IPTases modify both cytosolic and mitochondrial tRNAs and mitochondrial localization is a significant part of the biology. *S. cerevisiae* employs an intricate system for maintaining proper distribution of Mod5 to the mitochondria, nucleus and cytoplasm [27]–[29]. A prominent phenotype of *S. pombe tit1-Δ* mutants is slow growth on glycerol, a manifestation of mitochondrial respiratory dysfunction [10]. In *C. elegans*, the extended life span phenotype as well as deregulated development and other phenotypes of *gro-1* mutants can be rescued by the mitochondrial isoform of the GRO-1 IPTase but not the nuclear and cytoplasmic isoform [30]. It is therefore noteworthy that while both cytosolic and mitochondrial tRNAs are lacking i^6^A37 in patient cells, the manifestations of disease clearly localize to a mitochondrial cause. Therefore, it may be important that in addition to hypomodification of mt-tRNA^Ser(UCN)^, the overall levels of the mt-tRNA^Ser(UCN)^ were significantly lower in patient fibroblasts. This suggests that in addition to the ∼4-fold loss of tRNA specific activity due to lack of i^6^A37 [10], mitochondrial translation would be even further compromised by a decrease in the absolute level of mt-tRNA^Ser(UCN)^. This may contribute to a molecular basis for the apparent sensitization of the mitochondrial-associated phenotype in the patients described here.

Another informative finding was that the TRIT1-mutant could modify its substrate tRNAs with i^6^A37 when greatly over-expressed in the transduced patient fibroblasts. This was not completely unexpected on the basis of two experimental observations. As noted above, structure modelling suggested that the mutation would affect substrate binding but not i^6^A37 catalytic activity. Indeed, we observed increased transferase activity with increased substrate concentration, at least within the technical limits of the assay (Figures 3B and 3C). Second, the TRIT1 p.Arg323Gln mutant manifested partial activity to complement the slow growth in glycerol phenotype in the *S. pombe tit1-Δ* strain (Figure 3E). Furthermore, previous studies have shown that this phenotype remains uncomplemented by a prior characterized *tit1-T12A* catalytic mutant that is inactive for i^6^A37 modification of tRNA [10], [11]. Thus, the partial complementation of this phenotype (Figure 3E) suggests that the TRIT1 mutant retains some i^6^A37 activity, consistent with high activity of the *nmt1* ***^+^*** promoter in the multi-copy expression vector.

Recent studies have concluded that although some tRNAs in human cells contain the A36A37A38 TRIT1 recognition motif they accumulate in the i^6^A37-unmodified form [12]. Somewhat similarly, the A36A37A38-containing tRNA^Trp(CCA)^ in *S. cerevisiae* remains unmodified [11]. This further suggests that the subset of i^6^A37-containing tRNAs may change under different conditions, due to varying concentrations of the enzyme or substrate, a situation that may occur during development and/or other physiological conditions.

## Materials and Methods

### Ethics statement

Written informed consent was obtained from the family in accordance with the Declaration of Helsinki and the study was approved by the Newcastle and North Tyneside 1 Ethics Committee.

### Histochemical and biochemical analyses

Standard histological and histochemical analyses, including cytochrome *c* oxidase (COX), of a skeletal muscle biopsy were performed according to established protocols [31], on fresh-frozen skeletal muscle sections (10 µm). Mitochondrial respiratory chain complex activities were determined in skeletal muscle homogenates as previously described, and expressed relative to the activity of the matrix marker enzyme, citrate synthase [32].

### Molecular genetics

Total DNA was extracted by standard procedures from all available tissues obtained with consent from familial relatives, and mtDNA rearrangements were excluded by long-range PCR. Direct sequencing of the entire mitochondrial genome was performed on homogenate skeletal muscle DNA.

Genomic DNA from the two affected siblings (II–1 and II–3) was isolated from blood (DNeasy, Qiagen, Valencia, CA); fragmented to 150–200 bp with the use of Adaptive Focused Acoustics (Covaris); end-repaired, adenylated, and ligated to adapters (Illumina Paired-End Sample Preparation Kit). Ligated libraries were hybridized with whole-exome baits that covered 27,184 genes (Agilent SureSelect Human All Exon Kit Version 2) with modifications for the SureSelect Human All Exon Kit Illumina Paired-end Sequencing Library (Version 2.0.1). Captured fragments were purified, clonally amplified and sequenced on 2 lanes of an Illumina Genome Analyser IIx using 75 bp paired-end reads.

The sequence was aligned to the human reference genome (UCSC hg19) with Burrows Wheeler Aligner (BWA) [33], then reformatted with the use of SAMtools v0.1.18 [34]. 83.1% of exon target sequence was covered by >10 reads. Single base variants were identified with Varscan v2.2 [35] and Indels were identified with Dindel v1.01 [36]. Variants were annotated using wANNOVAR [37]. Lists of on-target variants were filtered against data from the National Heart, Lung and Blood Institute (NHLBI, NIH, Bethesda, MD) Exome Sequencing Project (ESP) 6500 exomes, the 1000 Genomes project, and the exome sequences of 315 unrelated in-house control exomes to identify rare homozygous variants with a Minor allele frequency (MAF) <0.01.

Variant filtering led to a final list of 40 rare, homozygous, protein-altering variants of which 4 were mitochondrial according to the Gene-Ontology database. These genes included *TRIT1, CCDC19, ARSB and SYNJ2* of which *TRIT1* segregated with disease in the family. Targeted resequencing and familial segregation studies were performed by cycle sequencing using an ABI 3130xl (Applied Biosystems) system and BigDye Terminator v3.1 technology. The following primer pairs, including universal tags, were employed: forward primer, 5′-TGTAAAACGACGGCCAGTAGGGAAAATGCACACTGGAG-3′, and reverse primer, 5′-CAGGAAACAGCTATGACCTTCCCTTAGGTCAGATCCAAAA-3′. Analysis of the evolutionary conservation of the mutated amino acid across a range of homologous proteins was performed by the freely available Clustal Omega multiple sequence alignment software (http://www.ebi.ac.uk/Tools/msa/clustalo/) [38].

### Culture of primary fibroblasts

Primary human fibroblast cell lines were established from the patient as well as from controls according to standard protocols and cultured at 37°C, in a humidified, 5% CO_2_ atmosphere. Fibroblasts were maintained as monolayers in Minimum Essential Media (MEM) (Life Technologies #21090) supplemented with FBS to 10%, 1x MEM-vitamins, 21 mM L-Glutamine, 1 mM sodium pyruvate, 1x penicillin/streptomycin, 1x non-essential amino acids, and 0.41 µM uridine.

### Fibroblast transduction

TRIT1 wild-type and p.Arg323Gln mutant open reading frames were cloned into the pOP retroviral vector (Radichev et al., 2006) in frame with FLAG and HA epitope tags at the 5′ end using the XhoI and NotI sites, and sequencing was performed for confirmation. The preparation of the retroviral supernatants and the transduction of the control and patient fibroblasts were done as described [39].

### Micro-scale oxygraphy analysis of live cells

Live cell respiration studies were performed by micro-scale oxygraphy using the Seahorse XF^e^ Extracellular Flux Analyzer 24 (Seahorse Bioscience) according to manufacturer's instructions. Fibroblasts were seeded at a density of 30,000 cells/well. Mitochondrial function was assayed through the sequential addition of oligomycin (to 1.3 µM) to block the ATP synthase, 2 additions of carbonyl cyanide 4-(trifluoromethoxy)-phenylhydrazone (FCCP), a respiratory uncoupler which drives maximal respiration (to 2 µM and then to 3 µM), and antimycin (to 2.5 µM) to inhibit Complex III.

Oxygen consumption rate (OCR) and proton production rate (PPR) measurements for each well were normalized by cell number. Non-mitochondrial respiration was subtracted from all OCR values prior to analysis; spare respiratory capacity (SRC) equals maximal OCR - basal OCR, ATP coupling efficiency equals (basal OCR - oligomycin-inhibited OCR)/(basal OCR*100). Seven separate control cell lines underwent multiple testing and the means were combined to calculate control data (mean ± SD; n = 7). Patient fibroblasts were tested multiple times (n = 21). An unpaired, two-tailed Student's t-test was performed to determine the significance of differences between the data sets and P-values were considered significant at the 95% confidence interval.

### SDS-PAGE and immunoblotting

Total cellular protein was extracted from patient and control fibroblasts (as well as transfected cell lines), size separated on a 10% separating gel by SDS-PAGE and transferred to a methanol-activated PVDF membrane. Immunoblotting was performed using primary antibodies to NDUFA9, NDUFB8, NDUFA13, SDHA, UQCRC2, MTCOI, MTCOII, COXIV and ATPB (all from Abcam), TRIT1 (GeneTex GTX120508) and β-actin (Sigma A5316) as a loading control and TOMM20 (Abcam) as a non-respiratory chain protein mitochondrial control. Chemiluminescent detection of the bands was achieved using the Amersham ECL Prime Kit (GE Healthcare) for signal development, following manufacturer's instructions and the membrane was viewed using the ChemiDoc^TM^MP Imaging System (Bio-Rad).

### Mitochondrial preparation and subfractionation

Subcellular fractions were prepared as described previously [40]. The same amount of protein (40 µg) from whole cell lysate, post-mitochondrial supernatant and mitochondrial subfractions was loaded onto a 12% SDS-PAGE gel, transferred to a PVDF membrane and analysed by immunoblotting using primary antibodies to TOMM20 (Santa Cruz), AIF (NEB), GDH (custom made against mature recombinant protein), NDUFA9 (Mitosciences), eIF4E (Cell Signaling). Chemiluminescent detection of the bands was achieved as described before.

### Metabolic labelling of *in vitro* mitochondrial translation

The translation of proteins encoded by the mtDNA in patient fibroblasts was assessed by labelling with ^35^S-methionine/^35^S-cysteine (Perkin Elmer) as described previously [41]. Cytosolic translation was inhibited by co-incubation of the radioisotopes with 100 µg/ml emetine dihydrochloride. Total cell protein was extracted from both control and patient fibroblasts and 50 µg loaded onto a 15%–20% gradient gel for SDS-PAGE. Assessment of protein loading was achieved by Coomassie blue staining, and the gel was visualised by exposure to a blank PhosphorImager screen that was imaged using a Typhoon system (GE Healthcare).

### *In vitro* modification assay

The *in vitro* modification activity of both wild-type and mutant TRIT1 was determined as previously described [11] using recombinantly-expressed enzyme from *E. coli* recovered using a Histidine tag [42]. Synthetic RNA minihelixes representing the target anticodon stem/loop (ASL) sequences of various tRNAs were used as templates for modification with ^14^C-labelled dimethylallyl pyrophosphate (DMAPP). In this assay, the isopentenyl group of DMAPP is transferred to A37 in substrate tRNAs by the IPTase TRIT1. The following RNA oligos were designed with an additional G-C base pair added to each ASL to stabilize the stem, and purchased from Integrated DNA Technologies (IDT): rGrUrGrCrArGrGrCrUrUrCrArArArCrCrUrGrUrArC (cy-tRNA^Sec(UCA)^), rGrArUrGrGrArCrUrUrGrArArArUrCrCrArUrC (cy-tRNA^Ser(UGA)^), rGrGrGrUrUrGrGrCrUrUrGrArArArCrCrArGrCrUrC (mt-tRNA^Ser(UCN)^), rGrGrGrUrUrGrGrCrUrUrGrArArGrCrCrArGrCrUrC (mt-tRNA^Ser(UCN)^-A7480G), rGrUrUrGrArArUrUrGrCrArArArUrUrCrGrArC (mt-tRNA^Cys^) and rGrUrArArArArCrUrUrArArArArCrUrUrUrArC (mt-tRNA^Leu(UUR)^).

Decreased hybridization efficiency of complimentary DNA oligos due to the incorporation of the isopentenyl group in synthetic RNA minihelixes was confirmed by running an *in vitro* modification reaction with unlabeled DMAPP and recombinant His-TRIT1. We used the same protocol for the *in vitro* modification reaction as described previously [11], replacing ^14^C-labeled DMAPP with unlabeled DMAPP (100 nmol). For each sample, two reactions were performed. In mock-treated samples, all the components of the *in vitro* reaction were added except His-TRIT1. After the reaction, the RNA sample was purified by a phenol-chloroform extraction and loaded on a 15% TBE-Urea gel. The RNA was transferred to a GeneScreen Plus Hybridization Transfer Membrane (Perkin Elmer, catalog # NEF986001PK) and hybridized with ^32^P-labeled anticodon loop (ACL) oligos as described in the legend of Figure 6D. The sequences of DNA oligos used for this experiment are the same as used elsewhere in this paper.

### The PHA6 tRNA-i^6^A37 detection assay

Total RNA isolation from skeletal muscle and human primary cell lines was performed using TRIzol according to manufacturer's protocol. The impact of the TRIT1 mutation on *in vivo* levels of the i^6^A37 modification in both cytosolic and mitochondrial tRNAs was assessed by the **P**ositive **H**ybridisation in the **A**bsence of i**^6^**A (PHA6) assay, which is an adaptation of high-resolution northern analysis [11]. The following anticodon loop (ACL) and body (BP) probes were used (all written as 5′-3′): mt-tRNA^Cys^ ACL, TCTTCGAATTTGCAATTCAATATG and BP, AGCCCCGGCAGGTTTGAAGCT, cy-tRNA^Ser(UGA)^ ACL, CCCATTGGATTT CAAGTCCAACGC, and BP, GCAGGATTCGAACCTGCGCGGG, wild-type mt-tRNA^Ser(UCN)^ ACL, CAAAGCTGGTTTCAAGCCAACCCC (used for analysis of the patient carrying the *TRIT1* mutation), mutant mt-tRNA^Ser(UCN)^ ACL, CAAAGCTGGCTTCAAGCCAACCCC (both the wild-type and mutation-bearing probes were used together as a ‘double ACL probe’; the complement of the mutated base is underlined) and mt-tRNA^Ser(UCN)^ BP, AAGGAAGGAATCGAACCCCCC, mt-tRNA^Leu(UUR)^ BP, GTTAAGAAGAGG AATTGAACCTC and U5 probe, TCCTCTCCACGGAAATCTTTA.

### Phenotype rescue analyses in *S. pombe*

The wild-type *TRIT1* or mutant *TRIT1* was cloned into the pREP82X plasmid under the *nmt1* ***^+^*** promoter before transformation into a yNB5 (tit1-Δ) strain of *S. pombe*. The experiments related to tRNA-mediated anti-suppression and growth deficiency in glycerol were performed as described previously [10], [11].

### Phenotype rescue analyses in *S. cerevisiae*

The *S. cerevisiae* yeast strain used was BY4742 *mod5-Δ* (*MATα his3Δ1 leu2Δ0 lys2Δ0 ura3Δ0 mod5::KanMX4*) from the Euroscarf collection. The *MOD5* gene was PCR-amplified with Kod HiFi Polymerase using primers MOD5CFw (gactagaaaatcgatgtgtcagg) and MOD5CSalIRv (ccgccGTCGACgcttgtcat cctccctttcc), digested with *Kpn*I and *Sal*I and cloned in the centromeric vector, pFL38 [43], thus obtaining the plasmid pFL38*MOD5*. The *mod5^K294R^* humanized and *mod5^K294Q^* mutant alleles were obtained by site-directed mutagenesis as described previously [44], on a *MOD5* gene fragment obtained through amplification with the upstream forward primer MOD5MUTFw (ggagcccctgcagcttcatg) and the reverse mutagenic primer MOD5hK294RFw (cgagaacacgtcaatacgcaCGCaggcaggtaaaatggatcaag) or MOD5K294QFw (cgagaacacgtc aatacgcaCaaaggcaggtaaaatggatcaag). The amplified fragments were digested with *Bam*HI and *Sal*I and subcloned in *Bam*HI-*Sal*I-digested pFL38*MOD5*.

Plasmids were introduced in a BY4742 *mod5Δ* strain according to [45]. Growth assays were performed at 28°C in SD medium (0.69% YNB (Formedium, Norfolk, UK), without amino acids for which the strain is auxotroph) supplemented with 2% glucose (w/v) or 2% ethanol (v/v). Images of the colonies in the spots were acquired at 40X magnification with a Zenith inverted microscope through an Optikam 3 Digital Camera (Optika).

Oxygen consumption rate was measured at 30°C from suspensions of yeast cells cultured for 24 hours at 28°C in SD medium supplemented with glucose at a non-repressing concentration of 0.6% using a Clark-type oxygen electrode (Oxygraph System Hansatech Instruments England) with 1 ml of air-saturated respiration buffer (0.1 M phthalate – KOH, pH 5.0), 0.5% glucose. The reaction was started by addition of 20 mg of wet-weight cells.

## Supporting Information

Figure S1 *MOD5-Δ S. cerevisiae* exhibit a mild respiratory defect that is not rescueable with mutant *Mod5^K294Q^*. **A**) A budding yeast *Saccharomyces cerevisiae* strain, *mod5-Δ*, was transformed with wild-type *MOD5*, an empty vector, a “humanized” *mod5^K294R^* allele or the mutant *mod5^K294Q^* allele. Calculation of the doubling times (mins) for each yeast strain grown on glucose (black columns) or ethanol (grey columns) confirmed an oxidative growth defect in *mod5-Δ* yeast transformed with an empty vector or a mutant *mod5^K294Q^* allele but normal growth in *mod5-Δ* yeast transformed with a humanized *mod5^K294R^* allele. *: p<0.05;**<0.01 (unpaired two-tailed t-test). **B**) Respiratory rates were normalized to the strain transformed with wild-type *MOD5*, for which the respiratory rate was 103 nmol.min^−1^.mg^−1^. The *mod5-Δ* yeast transformed with a ‘humanized’ *mod5^K294R^* allele showed normal respiration, whilst *mod5-Δ* yeast transformed with an empty vector or a mutant *mod5^K294Q^* allele had significantly reduced respiration rates. Values are the mean of three independent experiments, each with an independent clone. *: p<0.05;**<0.01:***: p<0.001 (paired two-tailed t- test). The error bars displayed on each graph indicate standard deviation.
(TIF)

Table S1 mtDNA sequencing data. Whole mtDNA sequencing of the proband (II-3) identified a number of known polymorphisms but no candidate pathogenic mutations (based upon the MitoMap (http://www.mitomap.org/MITOMAP) and mtDB (http://www.mtdb.igp.uu.se/) databases as well as our in-house database of >950 human mtDNA sequences). The non-coding region refers to the D-loop region of mtDNA, which is highly polymorphic and encodes no genes; the coding region refers to each of the 13 protein-encoding genes, 22 mt-tRNA genes and 2 mt-rRNA genes.
(DOCX)

Table S2 Variant numbers from in-house bioinformatic pipeline. On-target: Chromosome and position of variants matches within exome capture target coordinates +/− 500bp. Rare: Variant has a Minor Allele Frequency (MAF) less than 0.01 in the 1000 genomes, NHLBI-6500-ESP or 315 In-House exome databases. Protein Altering: Annovar predicts variant is ‘exonic’ or ‘splicing’, but excluding ‘synonymous’. Shared Homozygous: Variant genotype is homozygous (V/V) in one or both patient, allowing for non-coverage (0) in one patient. Mitochondrial (GO-terms): Gene containing variant is listed with the search term ‘mitoch*’ in the Gene-Ontology database. Mitochondrial (‘Original’ gene-list): Gene containing variant is listed as being ‘mitochondrial’ on Mootha gene list.
(DOCX)

Text S1 Clinical summary. A detailed report of the proband's clinical presentation and disease course is provided along with clinical information about his affected sister.
(DOCX)
