# Supplementary figures and images for: Defective i6A37 Modification of Mitochondrial and Cytosolic tRNAs Results from Pathogenic Mutations in TRIT1 and Its Substrate tRNA
Source: PLoS Genet. 2014 Jun 5;10(6):e1004424. doi: 10.1371/journal.pgen.1004424 (PMC4046958; doi:10.1371/journal.pgen.1004424)

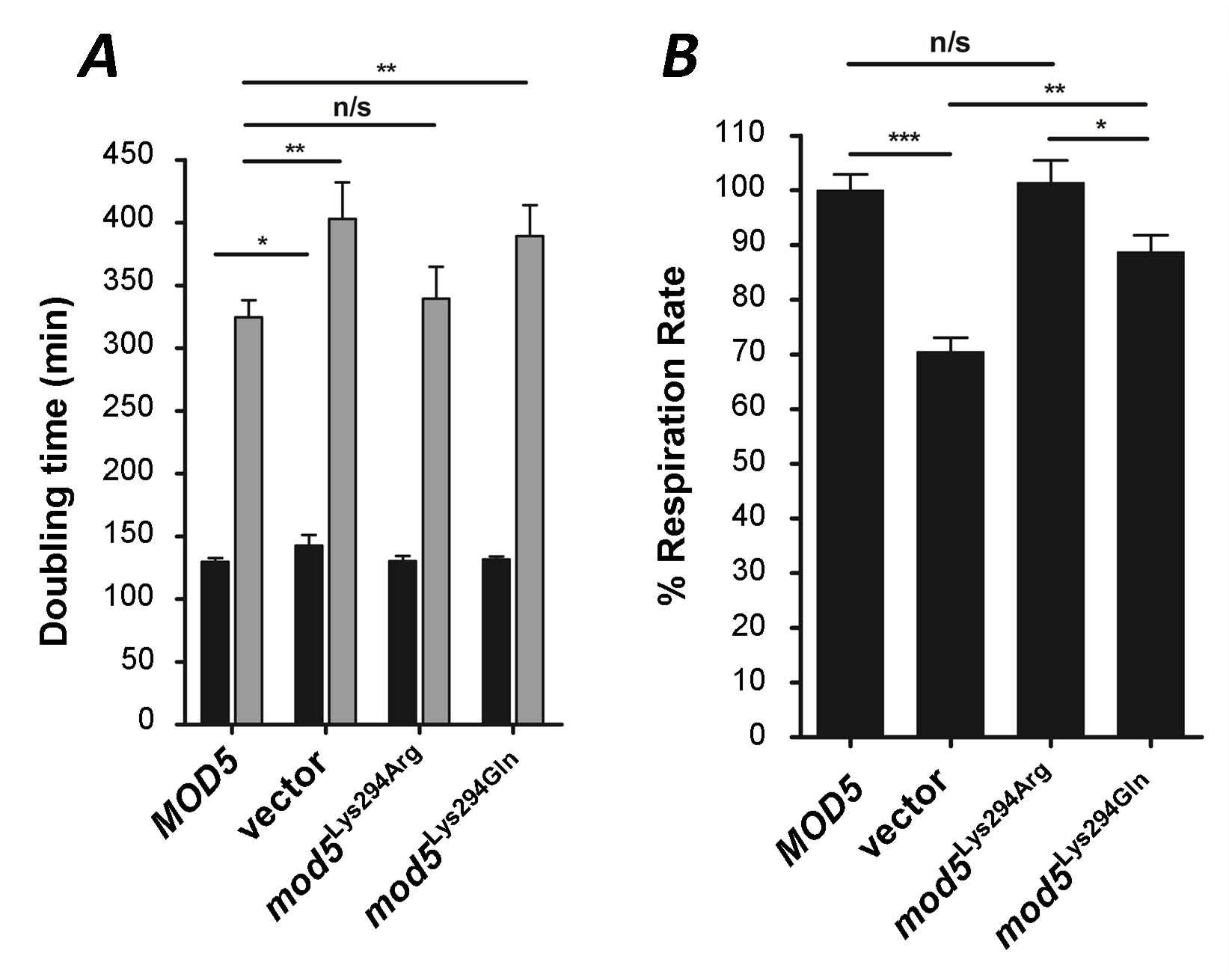

Supplement: Figure S1 — MOD5-Δ S. cerevisiae exhibit a mild respiratory defect that is not rescueable with mutant Mod5K294Q. A) A budding yeast Saccharomyces cerevisiae strain, mod5-Δ, was transformed with wild-type MOD5, an empty vector, a “humanized” mod5K294R allele or the mutant mod5K294Q allele. Calculation of the doubling times (mins) for each yeast strain grown on glucose (black columns) or ethanol (grey columns) confirmed an oxidative growth defect in mod5-Δ yeast transformed with an empty vector or a mutant mod5K294Q allele but normal growth in mod5-Δ yeast transformed with a humanized mod5K294R allele. *: p<0.05;**<0.01 (unpaired two-tailed t-test). B) Respiratory rates were normalized to the strain transformed with wild-type MOD5, for which the respiratory rate was 103 nmol.min−1.mg−1. The mod5-Δ yeast transformed with a ‘humanized’ mod5K294R allele showed normal respiration, whilst mod5-Δ yeast transformed with an empty vector or a mutant mod5K294Q allele had significantly reduced respiration rates. Values are the mean of three independent experiments, each with an independent clone. *: p<0.05;**<0.01:***: p<0.001 (paired two-tailed t- test). The error bars displayed on each graph indicate standard deviation. (TIF) [file pgen.1004424.s001.tif]
