# Supplementary material for: Defective i6A37 Modification of Mitochondrial and Cytosolic tRNAs Results from Pathogenic Mutations in TRIT1 and Its Substrate tRNA
Source: PLoS Genet. 2014 Jun 5;10(6):e1004424. doi: 10.1371/journal.pgen.1004424 (PMC4046958; doi:10.1371/journal.pgen.1004424)
Supplement: Table S1 — mtDNA sequencing data. Whole mtDNA sequencing of the proband (II-3) identified a number of known polymorphisms but no candidate pathogenic mutations (based upon the MitoMap (http://www.mitomap.org/MITOMAP) and mtDB (http://www.mtdb.igp.uu.se/) databases as well as our in-house database of >950 human mtDNA sequences). The non-coding region refers to the D-loop region of mtDNA, which is highly polymorphic and encodes no genes; the coding region refers to each of the 13 protein-encoding genes, 22 mt-tRNA genes and 2 mt-rRNA genes. (DOCX) [file pgen.1004424.s002.docx]

| **Non-coding region** | | **Coding region** | |
| --- | --- | --- | --- |
| **Mutation** |  | **Mutation** | **Gene** |
| m.73A>G | Non-coding region | m.750A>G | *MTRNR1* |
| m.146T>C | Non-coding region | m.1438A>G | *MTRNR1* |
| m.152T>C | Non-coding region | m.1811A>G | *MTRNR2* |
| m.234A>G | Non-coding region | m.1888G>A | *MTRNR2* |
| m.263A>G | Non-coding region | m.3106delC | *MTRNR2* |
| m.311insC | Non-coding region | m.4796A>G | *MTND2* |
|  |  | m.5186A>T | *MTND2* |
| m.16051A>G | Non-coding region | m.6629A>G | *MTCO1* |
| m.16209T>C | Non-coding region | m.7028C>T | *MTCO1* |
| m.16239C>T | Non-coding region | m.8860A>G | *MTATP6* |
| m.16352T>C | Non-coding region | m.9094C>T | *MTATP6* |
| m.16353C>T | Non-coding region | m.9614A>G | *MTCO3* |
| m.16519T>C | Non-coding region | m.11467A>G | *MTND4* |
|  |  | m.11719G>A | *MTND4* |
|  |  | m.12106C>T | *MTND4* |
|  |  | m.12308A>G | *MTTL2* |
|  |  | m.12372G>A | *MTND5* |
|  |  | m.12793T>C | *MTND5* |
|  |  | m.13194G>A | *MTND5* |
|  |  | m.13656T>C | *MTND5* |
|  |  | m.14766C>T | *MTCYB* |
|  |  | m.15049C>T | *MTCYB* |
|  |  | m.15326A>G | *MTCYB* |
|  |  | m.15326A>G | *MTCYB* |
|  |  | m.15930G>A | *MTTT* |
