# Supplementary material for: Defective i6A37 Modification of Mitochondrial and Cytosolic tRNAs Results from Pathogenic Mutations in TRIT1 and Its Substrate tRNA
Source: PLoS Genet. 2014 Jun 5;10(6):e1004424. doi: 10.1371/journal.pgen.1004424 (PMC4046958; doi:10.1371/journal.pgen.1004424)
Supplement: Table S2 — Variant numbers from in-house bioinformatic pipeline. On-target: Chromosome and position of variants matches within exome capture target coordinates +/− 500bp. Rare: Variant has a Minor Allele Frequency (MAF) less than 0.01 in the 1000 genomes, NHLBI-6500-ESP or 315 In-House exome databases. Protein Altering: Annovar predicts variant is ‘exonic’ or ‘splicing’, but excluding ‘synonymous’. Shared Homozygous: Variant genotype is homozygous (V/V) in one or both patient, allowing for non-coverage (0) in one patient. Mitochondrial (GO-terms): Gene containing variant is listed with the search term ‘mitoch*’ in the Gene-Ontology database. Mitochondrial (‘Original’ gene-list): Gene containing variant is listed as being ‘mitochondrial’ on Mootha gene list. (DOCX) [file pgen.1004424.s003.docx]

|  | **On-Target SNVs** | **On-Target Indels** | **Protein Altering (SNV/Indel)** | **Shared Homozygous, Protein Altering** | **Rare (MAF<0.01)** | **Mitochondrial (GO-terms)** | **Mitochondrial (‘Original’ gene list)** |
| --- | --- | --- | --- | --- | --- | --- | --- |
| **Patient II-1** | 43,248 | 2,170 | 9,971 | 3,970 | 40 | 4 | 1 |
| **Patient II-3** | 43,568 | 2,166 | 10,098 |  |  |  |  |
