# Supplementary material for: Defective i6A37 Modification of Mitochondrial and Cytosolic tRNAs Results from Pathogenic Mutations in TRIT1 and Its Substrate tRNA
Source: PLoS Genet. 2014 Jun 5;10(6):e1004424. doi: 10.1371/journal.pgen.1004424 (PMC4046958; doi:10.1371/journal.pgen.1004424)
Supplement: Text S1 — Clinical summary. A detailed report of the proband's clinical presentation and disease course is provided along with clinical information about his affected sister. (DOCX) [file pgen.1004424.s004.docx]

Clinical Report

The male proband (II-3) is the third child of consanguineous, UK-Pakistani parents. Pregnancy was complicated by maternal diabetes mellitus and pregnancy induced hypertension and, as a result of the latter, he was delivered at 35 weeks gestation by emergency Cesarean section. He did not require any postnatal resuscitation. Birth weight at 3060 g was on the 91^st^ centile while head circumference at 32cm was just below the 50^th^. During the first year of life his mother noted intermittent jerking of his limbs and head nods, but it was not until the age of 15 months (13.5 months corrected) that he came to medical attention following a febrile convulsion. At this time microcephaly and global neurodevelopmental delay were noted; neurometabolic and genetic investigations were initiated, but no cause identified. Over the next 18 months he experienced recurrent seizures, initially febrile, but subsequently non-febrile, and was commenced on Carbamazepine; later changed to Sodium Valproate when myoclonic seizures became evident. Seizures remained well controlled for approximately 5 years on this anticonvulsant, but following a number of severe, prolonged seizures at age 7 years, his medication was revised to a combination of Vigabatrin and Clonazepam, with some benefit for seizure control. Cranial MRI performed at age 7 yr was diagnostically unhelpful, but EEG confirmed a myoclonic epilepsy on a background of severe cerebral impairment. Neurodevelopmental problems persisted, with delay in ambulation (walking at 3 years), expressive (and to a lesser extent receptive) language and social skills. Severe learning difficulties triggered the instigation of special schooling arrangements. No problems were identified with his vision or hearing.

At age 10 years the proband was admitted to hospital with a 3-week history of polyuria, polydipsia and nocturia. He was obese (47.3 kg) and had a blood glucose of 20 mmol/L with both glucose and ketones present in urine. A diagnosis of Diabetes Mellitus was made and he commenced insulin, on which he remains. Myoclonic jerks re-emerged over the next few years and, in the context of his other problems, this prompted referral to a specialist Mitochondrial Disease Clinic in Newcastle-upon-Tyne, UK. When assessed at age 16 years, his mother reported him to be excessively sleepy. On examination he was uncooperative and somewhat aggressive. He was obese, microcephalic and had fragmentary myoclonic jerks of all 4 limbs accompanied by frequent head nods. He had no evidence of muscle weakness or cardiomyopathy. Treatment with Levetiracetam improved his myoclonic jerks and somnolence, suggesting that he had been experiencing a degree of epileptic encephalopathy. Repeat cranial MRI revealed a structurally normal, albeit small, brain. A skeletal muscle biopsy was performed following informed ethical consent from his mother. His clinical course has since stabilised though continued weight gain has led to hypertension and the development of non-alcoholic fatty liver disease.

The proband has an older sister (II-1) with very similar but less severe symptoms, an older brother (II-2) with diabetes mellitus (non-insulin dependent) but normal intelligence and consanguineous parents (I-1 and I-2), both of whom have diabetes mellitus. Several members of the extended family also have diabetes mellitus, some requiring insulin treatment.
